# Redox Responses in Patients with Sepsis: High Correlation of Thioredoxin-1 and Macrophage Migration Inhibitory Factor Plasma Levels

**DOI:** 10.1155/2010/985614

**Published:** 2010-08-05

**Authors:** Thorsten Brenner, Claudia Rosenhagen, Jochen Steppan, Christoph Lichtenstern, Jürgen Weitz, Thomas Bruckner, Eike O. Martin, Ursula Hoffmann, Markus A. Weigand, Stefan Hofer

**Affiliations:** ^1^Department of Anaesthesiology, University of Heidelberg, Im Neuenheimer Feld 110, 69120 Heidelberg, Germany; ^2^Department of Anaesthesiology and Critical Care Medicine, Johns Hopkins Medical Institutions, 600 N Wolfe Street, Baltimore, MD 21287, USA; ^3^Department of Anaesthesiology and Intensive Care Medicine, University of Giessen, Rudolf-Buchheim-Straße 7, 35392 Giessen, Germany; ^4^Department of Surgery, University of Heidelberg, Im Neuenheimer Feld 110, 69120 Heidelberg, Germany; ^5^Institute of Medical Biometry and Informatics, University of Heidelberg, Im Neuenheimer Feld 305, 69120 Heidelberg, Germany; ^6^Department of Medicine, Faculty of Clinical Medicine, University of Mannheim, Theodor-Kutzer-Ufer 1-3, 68167 Mannheim, Germany

## Abstract

*Background*. Redox active substances (e.g., Thioredoxin-1, Macrophage Migration Inhibitory Factor) seem to be central hubs in the septic inflammatory process. *Materials and Methods*. Blood samples from patients with severe sepsis or septic shock (*n* = 15) were collected at the time of sepsis diagnosis (*t*0), and 24 (*t*24) and 48 (*t*48) hours later; samples from healthy volunteers (*n* = 18) were collected once; samples from postoperative patients (*n* = 28) were taken one time immediately after surgery. In all patients, we measured plasma levels of IL-6, TRX1 and MIF. *Results*. The plasma levels of MIF and TRX1 were significantly elevated in patients with severe sepsis or septic shock. Furthermore, TRX1 and MIF plasma levels showed a strong correlation (*t*0: *r*_sp_ = 0.720, *ρ* = 0.698/*t24*: *r*_sp_ = 0.771, *ρ* = 0.949). *Conclusions*. Proinflammatory/~oxidative and anti-inflammatory/~oxidative agents show a high correlation in order to maintain a redox homeostasis and to avoid the harmful effects of an excessive inflammatory/oxidative response.

## 1. Introduction

Severe sepsis, septic shock, and the resulting multiple organ failure/dysfunction syndrome represent an ongoing challenge in intensive care units [1–5]. With mortality ranging from 40% to 70%, septic shock is the most common cause of death in intensive care medicine [2, 6]. Despite intensive basic research and clinical studies, the pathophysiology of sepsis is still poorly understood. Inflammation leads to oxidative stress because of the production of reactive oxygen species (ROS). Oxidative stress is a major contributing factor to the high mortality rates associated with several diseases such as endotoxic shock. Immune cells therefore need adequate levels of antioxidant defenses in order to avoid harmful effects of an excessive ROS production and to keep a well-balanced redox homeostasis. In this context, two substances have recently become of great interest: (1) Macrophage Migration Inhibitory Factor (MIF), a proinflammatory protein, which is released by immune cells and shows elevated levels in sepsis syndrome, as well as (2) Human Thioredoxin-1 (TRX1), a potent antioxidant that modulates inflammation, cell growth, and apoptosis, which seems to counteract the proinflammatory and pro-oxidant effects of MIF [7–9]. Therefore, these two biomarkers appear to be central hubs in the inflammatory setting and are therefore of great interest. However, no sufficient knowledge exists about the role of these key mediators in severe sepsis or septic shock. The aims of this study were therefore twofold: (1) to assess the plasma levels of each parameter in different inflammatory settings (healthy volunteers, postoperative, and septic patients) and (2) to establish whether plasma levels of TRX1 and MIF correlate with each other.

## 2. Materials and Methods

The observational clinical study was approved by the local ethics committee and was conducted in the surgical and medical intensive care units of the University Hospitals of Heidelberg and Mannheim, Germany. All study and control patients or their legal designees signed written informed consent. In total, 61 individuals in three groups were enrolled in the study (Table 1). The three groups included 15 patients with severe sepsis or septic shock (referred to as the septic group), 28 patients after major abdominal surgery (the postoperative group), and 18 healthy volunteers (the volunteer group). The 15 patients were classified as having severe sepsis or septic shock based on the criteria of the International Sepsis Definitions Conference [10]. Patients were eligible for enrollment with an onset of sepsis syndrome ≤24 hours. The initial blood draw was also performed within this period. In contrast, patients with an onset of sepsis syndrome >24 hours were excluded from the study. The management of patients with severe sepsis or septic shock in the intensive care unit included early goal-directed therapy (according to Rivers and colleagues [11]), elimination of the septic focus, and broad-spectrum antibiotics [12, 13]. The second group included 28 patients undergoing major abdominal surgery, with negative parameters for systemic inflammatory response syndrome (Table 1). As a control group, we chose 18 healthy young volunteers without any signs of infection (Table 1). Blood samples from patients with severe sepsis were collected within 24 hours after the diagnosis of sepsis, and also 24 and 48 hours later. In the septic group, severity of illness was estimated using the Acute Physiology and Chronic Health Evaluation (APACHE) II score as well as the Sequential Organ Failure Assessment (SOFA) score and the Simplified Acute Physiology Score (SAPS) II. Patients with sepsis were re-evaluated for survival 90 days after enrollment in the study. This evaluation was performed using available hospital records. In case of the patient's discharge from the hospital, the family doctor was contacted. If necessary, we furthermore got in contact with the patient himself. Blood samples from the postoperative group were collected once immediately after surgery, and the samples from the volunteer group were taken one time. After blood collection, plasma of all study participants was immediately obtained by centrifugation, transferred into cryotubes, and stored at −80°C until further processing. Human Interleukin-6 (IL-6) was measured in order to determine the ongoing inflammatory response. Furthermore, the activation of the redox system was evaluated. Therefore, we measured plasma levels of Human Thioredoxin-1 (TRX1) and Macrophage Migration Inhibitory Factor (MIF). We used ELISA kits to determine plasma concentrations of Interleukin-6 (IL-6, R&D Systems, Minneapolis, MN, USA), Human Thioredoxin-1 (Human TRX1, LabFrontier Co., Ltd, Seoul, Korea), and Macrophage Migration Inhibitory Factor (Human MIF, RayBiotech, Inc., Norcross, GA, USA) according to the manufacturers' instructions.

All assays were performed in duplicate. The resulting study data were entered into an electronic database (Microsoft Excel 2002, Microsoft Corporation, Redmond, WA, USA) and evaluated using SPSS software (Statistical Product and Services Solutions, Version 16.0, SPSS Inc, Chicago, IL, USA). Categorical data were summarized by means of absolute and relative frequencies (counts and percentages). Quantitative data were summarized using the number of observations, mean and standard deviation, minimum, median with quartiles, or differences of the quartiles and maximum. Wherever appropriate, data were visualized using box-and-whisker plots. The Kolmogorov-Smirnov test was applied to check for normal distribution. Due to abnormally distributed data, nonparametric methods for evaluation were used (chi-square test for categorical data, Mann-Whitney *U*-test as well as Wilcoxon test for continuous data). Correlation analysis was performed using two-sided Spearman's rank correlation test as well as Pearson's product-moment correlation test. A *P* value < .05 was considered statistically significant. Concerning symbolism and higher orders of significance: *P* < .05:*, *P* < .01:**, *P* < .001:***.

## 3. Results

Age and sex of patients in the septic (61 years; 9 male sex) and postoperative (62 years; 15 male sex) groups were comparable (Table 1). In the septic group, patients who survived or died showed no significant differences in their demographic data (data not shown). In contrast, healthy volunteers (35 years; 10 male sex) were significantly younger compared with the septic and postoperative groups (Table 1). In the septic group, 8 of 15 patients (53.3%) survived (Table 1). No one in the postoperative or volunteer groups died during the study. The primary site of infection in the septic group was the gastrointestinal tract (6 patients, 40.0%). Furthermore, the septic focus was found to be in the respiratory tract (3 patients, 20%) or dedicated as a surgical complication (3 patients, 20%) (Table 1). A positive culture from the site of infection was obtained in 67% of all septic patients. In these patients, cultures were found to be gram-negative in 70% and gram-positive in 30%. Patients in the postoperative group primarily underwent surgery of the pancreas, whereas surgeries of the colon, liver, and the genitourinary tract were less frequent (Table 1).

**Figure 1:**
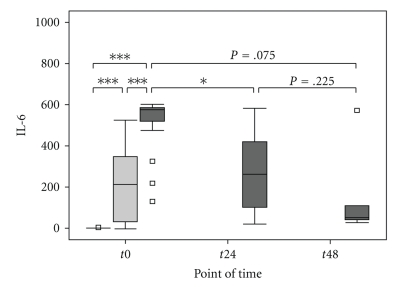
Comparison of Interleukin-6 (IL-6) in the volunteer, postoperative, and septic groups at baseline and at 24 and 48 hours in the septic group. Concentrations of Interleukin-6 (IL-6; (pg/ml)) were measured from the sera of healthy volunteers (“Healthy”, *n* = 18, white box), postoperative patients after major abdominal surgery (“Post-OP”, *n* = 28, light grey box), and patients with sepsis (“Sepsis”, *n* = 15, dark grey box), at *t*0 (measured once for the volunteer group, immediately after surgery for the postoperative group, and at the time of diagnosis of sepsis for the sepsis group). In addition, for the septic group, the 2 other times of data collection are represented, *t*24 and *t*48 for 24 and 48 hours, respectively, after the diagnosis of sepsis. Data in box plots are given as median, 25th percentile, 75th percentile, and the 1.5 interquartile range. Outliers are shown in form of circles (1.5–3 interquartile ranges above 75th percentile or below 25th percentile) or rectangles (>3 interquartile ranges above 75th percentile or below 25th percentile). Abbreviations: IL-6, Interleukin-6.

**Figure 2:**
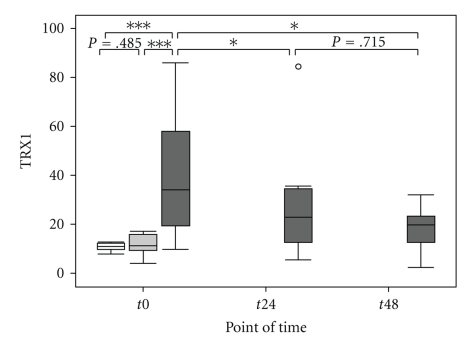
Comparison of Thioredoxin-1 (TRX1) measurements in the volunteer, postoperative, and septic groups at baseline and at 24 and 48 hours in the septic group. Concentrations of Thioredoxin-1 (TRX1; (ng/ml)) were measured from the sera of healthy volunteers (“Healthy,” *n* = 18, white box), postoperative patients after major abdominal surgery (“Post-OP,” *n* = 28, light grey box), and patients with sepsis (“Sepsis,” *n* = 15, dark grey box), at *t*0 (measured once for the volunteer group, immediately after surgery for the postoperative group, and at the time of diagnosis of sepsis for the sepsis group). In addition, for the septic group, the two other times of data collection are represented, *t*24 and *t*48 for 24 and 48 hours, respectively, after the diagnosis of sepsis. Data in box plots is given as median, 25th percentile, 75th percentile, and the 1.5 interquartile range. Outliers are shown in form of circles (1.5-3 interquartile ranges above 75th percentile or below 25th percentile) or rectangles (>3 interquartile ranges above 75th percentile or below 25th percentile). Abbreviations: TRX1, Thioredoxin-1.

**Figure 3:**
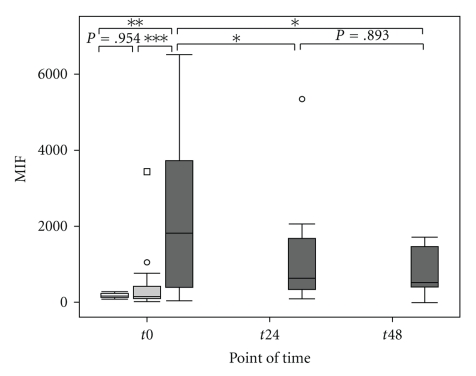
Comparison of Macrophage Migration Inhibitory Factor (MIF) measurements in the volunteer, postoperative, and septic groups at baseline and at 24 and 48 hours in the septic group. Concentrations of Macrophage Migration Inhibitory Factor (MIF; (pg/ml)) were measured from the sera of healthy volunteers (“Healthy,” *n* = 18, white box), postoperative patients after major abdominal surgery (“Post-OP,” *n* = 28, light grey box), and patients with sepsis (“Sepsis,” *n* = 15, dark grey box), at *t*0 (measured once for the volunteer group, immediately after surgery for the postoperative group, and at the time of diagnosis of sepsis for the sepsis group). In addition, for the septic group, the two other times of data collection are represented, *t*24 and *t*48 for 24 and 48 hours, respectively, after the diagnosis of sepsis. Data in box plots is given as median, 25th percentile, 75th percentile, and the 1.5 interquartile range. Outliers are shown in form of circles (1.5–3 interquartile ranges above 75th percentile or below 25th percentile) or rectangles (>3 interquartile ranges above 75th percentile or below 25th percentile). Abbreviations: MIF, Macrophage Migration Inhibitory Factor.

Septic patients were considered to be severely injured during the entire study period, as assessed by the APACHE II, SOFA, and SAPS II score, but showed no significant differences between the surviving and nonsurviving subgroups of septic patients (Table 2). Plasma levels of IL-6 were significantly elevated at the onset of sepsis compared with the postoperative and the volunteer groups (Figure 1 and Table 3). Furthermore, plasma levels of IL-6 were significantly elevated in the postoperative group compared with healthy volunteers (Figure 1 and Table 3). In the septic group, the level of IL-6 decreased significantly within 24 hours after sepsis onset (*t*0 → *t*24: *P* = .021*, *t*24 → *t*48: *P* = .225, *t*0 → *t*48: *P* = .075), but still remained significantly higher than the volunteer group (*t*24: *P* < .001***, *t*48: *P* < .001***) (Figure 1). Il-6 levels did not differ between the surviving and nonsurviving subgroup of septic patients at any time (Table 2). The plasma levels of TRX1 were significantly elevated at the time of diagnosis of sepsis, compared with levels in the postoperative and volunteer groups (Figure 2 and Table 3). TRX1 plasma levels decreased significantly within 48 hours after sepsis onset (*t*0 → *t*24 : *P* = .046*,  *t*24 → *t*48 : *P* = .715,  *t*0 → *t*48 : *P* = .028*), but still remained significantly elevated than the volunteer group (*t*24 : *P* = .114, *t*48 : *P* = .042*). In comparison to the postoperative group, TRX1 plasma levels of septic patients failed scarcely to show a significant difference at *t*24, as well as *t*48 (*t*24 : *P* = .061,  *t*48 : *P* = .069) (Figure 2). TRX1 plasma levels did not differ between the postoperative and volunteer groups (TRX1: *P* = .458) (Figure 2 and Table 3). Furthermore, between the surviving and nonsurviving subgroups of septic patients, TRX1 plasma levels did not show any significant difference (Table 2). The plasma levels of MIF were significantly elevated at the time of diagnosis of sepsis, compared with levels in the postoperative and volunteer groups (Figure 3 and Table 3). MIF plasma levels decreased significantly within 48 hours after sepsis onset (*t*0 → *t*24: *P* = .050*, *t*24 → *t*48: *P* = .893, *t*0 → *t*48: *P* = .028*), but still remained significantly elevated than the volunteer group (*t*24: *P* = .030*, *t*48: *P* = .048*) and the postoperative group (*t*24: *P* = .023*, *t*48: *P* = .069) (Figure 3). MIF plasma levels did not differ between the postoperative and volunteer groups (MIF: *P* = .954) (Figure 3 and Table 3). Furthermore, between the surviving and nonsurviving subgroups of septic patients, MIF plasma levels did not show any significant difference (Table 2).

**Figure 4:**
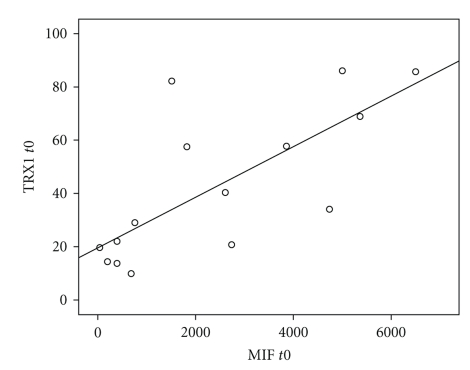
Strong correlation between TRX1 and MIF plasma levels in patients with severe sepsis or septic shock at the onset of sepsis syndrome. The scatter plot visualizes concentrations of Macrophage Migration Inhibitory Factor (MIF; (pg/ml)) as well as accompanying concentrations of Thioredoxin-1 (TRX1; (ng/ml)) from patients with severe sepsis or septic shock at the onset of sepsis syndrome (*t*0) (“Sepsis,” *n* = 15, black circles). Abbreviations: MIF, Macrophage Migration Inhibitory Factor; TRX1, Thioredoxin-1.

A correlation analysis using two-sided Spearman's rank correlation test, as well as Pearson's product-moment correlation test, indicated a strong correlation between TRX1 and MIF plasma levels in patients with severe sepsis or septic shock especially at the onset of sepsis syndrome (*t*0: *r*_sp_ = 0.720, *ρ* = 0.698) and 24 hours later (*t*24: *r*_sp_ = 0.771, *ρ* = 0.949) (Figure 4). In contrast, between TRX1 and IL-6 plasma levels as well as between MIF and IL-6 plasma levels in septic patients, no significant correlations could be observed at the onset of sepsis (TRX/IL-6, *t*0: *r*_sp_ = 0.143, *ρ* = 0.106; MIF/IL-6, *t*0: *r*_sp_ = 0.029, *ρ* = 0.127) and 24 hours later (TRX/IL-6, *t*24: *r*_sp_ = 0.107, *ρ* = 0.087; MIF/IL-6, *t*24: *r*_sp_ = 0.310, *ρ* = 0.232).

## 4. Discussion

The present study demonstrates that proinflammatory/~oxidative (Macrophage Migration Inhibitory Factor, MIF) as well as anti-inflammatory/~oxidative (Human Thioredoxin-1, TRX1) agents are significantly raised in patients with severe sepsis and septic shock. Positive correlations between these two mediators may suggest a linked role in the pathophysiology of sepsis.

Severe sepsis as well as septic shock and related multiple organ dysfunction syndrome is still the most common cause of death in intensive care medicine [1–6]. Many of the pathophysiological changes during sepsis are related to inflammation [14]. Not surprisingly, different markers of systemic inflammation (e.g., IL-6) and mediators involved in the redox homeostasis (e.g., TRX1, MIF) are significantly elevated during ongoing sepsis [15], whereas only IL-6 differed between patients after major abdominal surgery and healthy volunteers. This reflects generalized infection during sepsis, while patients after major abdominal surgery experience only a mild activation of their inflammatory system [16]. IL-6 is released into the bloodstream after any kind of tissue insult [17]. Therefore, IL-6 is the most commonly described cytokine after surgery. Concentrations of IL-6 peak 24 hours after the surgical procedure and return to the baseline value in a few days in noninfected patients [18]. In accordance to our investigation, IL-6 peak concentrations are reported to be generally in the range of 200–300 pg/ml [19]. The highest IL-6 serum/plasma concentrations are reached during sepsis, therefore it appears to be a good marker of the severity of infection [20, 21]. (Human) Thioredoxin-1 (TRX1) is an anti-inflammatory agent, whose anti-inflammatory effects are not yet completely understood. TRX1 is a redox-sensitive molecule that has pleiotropic cellular effects, functioning as a protective cellular antioxidant and regulator of transcription factor activity [22–27]. Together with the TRX-reductase and NADPH, TRX1 represents a major intracellular reducing agent containing a redox-active disulfide/dithiol within the conserved active site sequence Cys-Gly-Pro-Cys, and therefore protecting cells from oxidative stress [22–27]. Beside these antioxidative effects, TRX1 plays an important role in the modulation of the immune system by regulating DNA binding of several transcription factors (e.g., p53, NFkappaB, activator protein-1) [28–30]. Several investigations have shown that extracellular levels of TRX1 are increased in conditions of oxidative stress and inflammation [7, 30–39]. In accordance to these investigations, the present study was able to demonstrate that plasma TRX1 levels were significantly higher in patients with sepsis compared to healthy volunteers and postoperative patients. The elevated plasma levels of TRX1 then most likely reflect the increased oxidative stress in septic patients. In the literature, it was demonstrated that increased TRX1 plasma levels induced resistance to harmful conditions in animal models (e.g., LPS-induced acute hepatitis, cecal ligation, and puncture) [40–47] due to the ability of TRX1 to relieve local oxidative stress and to modulate neutrophiles extravasation into sites of inflammation [48]. Furthermore, TRX1 seems to be able to counteract the proinflammatory and pro-oxidant effects of Macrophage Migration Inhibitory Factor (MIF) [49].

MIF is another member of the TRX superfamily, but functions as a classical proinflammatory cytokine [9]. Macrophage Migration Inhibitory Factor (MIF) activates T-cells and macrophages, amplifies the production of other proinflammatory cytokines, and upregulates the expression of Toll-Like Receptor-4 (TLR-4) by phagocytes [9]. Because of the prevention of p53-dependent apoptosis of activated macrophages, high concentrations of MIF result in sustained inflammatory responses. Therefore, MIF seems to counteract the antioxidative effects of TRX. Accordingly, we were also able to demonstrate significantly higher MIF-levels in patients of the septic group in comparison to patients of the postoperative or volunteer groups. MIF is secreted by leukocytes, and its synthesis is induced by bacterial endo- and exotoxins and several proinflammatory mediators (Tumor Necrosis Factor-*α*/TNF-*α*, Interferon-*γ*/IFN-*γ*, Complement Factor 5a/C5a). For the acute phase of sepsis, it has been demonstrated that high plasma levels are harmful and correlate with sepsis severity [8]. The neutralization of MIF results in an attenuation of the inflammatory response and improved survival in experimental sepsis [8, 50]. In the light of these observations, the relationship between TRX1 and MIF in sepsis is of great interest. To our knowledge, only little work has been done so far in simultaneously determining plasma levels of TRX1 and MIF in human sepsis or septic animal models [49]. In accordance to the results of Leaver and colleagues [51], we were able to show raised plasma levels of TRX1 and MIF in patients with SIRS/sepsis, whereby plasma levels of TRX1 and MIF showed a unique correlation. Unfortunately, neither TRX1 nor MIF showed significant differences between the surviving and nonsurviving subgroups of the septic patients and therefore could not be used as an early predictor for survival in patients with severe sepsis or septic shock. This may be due to the small cohort of septic patients, so a re-evaluation in a larger cohort of septic patients needs to be performed. Furthermore, the detailed mechanisms of interaction between TRX1 and MIF are not yet completely understood. Son et al. recently suggested that cell surface TRX1 serves as one of the MIF binding molecules or MIF-receptor components and inhibits MIF-mediated inflammatory signals [52]. As a possible limitation of this investigation, the significant lower age of healthy volunteers in comparison to the postoperative group as well as the septic group has to be stated. At last, we are not able to estimate exactly the influence of patients' age on the redox marker measurements in the different study groups. Unfortunately, many previously published investigations dealing with these parameters are afflicted with the same problem [47, 51]. Nevertheless, it remains unlikely that the factor age might have considerably influenced the presented study results, since TRX1 and MIF plasma levels showed significant differences in patients of the same age (postoperative cohort versus septic cohort) and were comparable in patients of significant different ages (healthy volunteers versus postoperative cohort). Due to the positive correlations of TRX1 and MIF, our investigation supports a linked role of these two mediators in the pathophysiology of sepsis. This has important implications for further research on the pathogenesis of severe sepsis or septic shock.

## 5. Conclusions

In summary, our results suggest that substances involved in the redox homeostasis (e.g., TRX1, MIF) represent central hubs in the septic inflammatory response, as assessed by significantly elevated plasma levels of these mediators in patients with severe sepsis or septic shock. Proinflammatory/~oxidative and anti-inflammatory/~oxidative agents show a high correlation in order to maintain a redox homeostasis and to avoid harmful effects of an excessive inflammatory/oxidative response. For this reason, the detailed mechanisms of the TRX1 and MIF interaction, as well as their use as possible targets for therapeutic manipulation, represent areas for further research.

## Figures and Tables

**Table 1:** Baseline data of 15 patients in the septic group, 28 patients in the postoperative group, and 18 individuals in the volunteer group.

Septic group	
Demographic data	
Age, y	61.0 ± 13.7
Male sex	9 (60.0%)
Primary site of infection / septic focus	
Lung	3 (20.0%)
Gastrointestinal tract	6 (40.0%)
Genitourinary tract	1 (6.7%)
Surgical site	3 (20.0%)
Other	2 (13.3%)
Outcome	
Survivor	8 (53.3%)

Postoperative group	

Demographic data	
Age, y	62.3 ± 14.2
Male sex	15 (53.6%)
Primary site of surgery	
Pancreas	13 (46.4%)
Colon	5 (17.9%)
Liver	2 (7.1%)
Genitourinary	3 (10.7%)
Other abdominal	5 (17.9%)

Volunteer group	

Demographic data	
Age	34.5 ± 8.6
Male sex	10 (55.6%)

Data are presented by number (%), except for age (mean ± standard deviation).

**Table 2:** Comparison of IL-6, TRX1, and MIF plasma levels, as well as APACHE II, SAPS II, and SOFA scores in survivors and nonsurvivors in the septic group at baseline and at 24 and 48 hours.

		Survivor (*n* = 8)	Nonsurvivor (*n* = 7)	*P*-value
IL-6 (pg/ml)	*t*0	578.7; 451.4–579.9	576.1; 521.9–581.6	>.05
	*t*24	262.2; 103.4–300.3	371.2; 153.6–553.7	>.05
	*t*48	53.0; 41.0–79.6	51.7; 47.4–312.0	>.05
*P*-value	*t*0-*t*24-*t*48	>.05	>.05	

TRX1 (ng/ml)	*t*0	26.8; 14.2–44.7	57.5; 22.0–82.2	>.05
	*t*24	25.8; 13.4–47.8	22.9; 16.3–28.0	>.05
	*t*48	12.9; 9.5–16.1	23.8; 21.1–27.9	>.05
*P*-value	*t*0-*t*24-*t*48	>.05	>.05	

MIF (pg/ml)	*t*0	1645.8; 345.1–4078.7	1821.1; 971.9–3147.6	>.05
	*t*24	624.7; 396.3–1899.5	889.0; 432.4–1467.3	>.05
	*t*48	421.8; 315.3–692.7	951.1; 425.8–1469.0	>.05
*P*-value	*t*0-*t*24-*t*48	>.05	.05*	

APACHE II (points)	*t*0	43.0; 35.0–45.0	33.0; 32.5–37.0	>.05
	*t*24	35.0; 29.5–40.8	43.0; 38.8–45.0	>.05
	*t*48	34.0; 23.5–38.5	35.0; 35.0–36.0	>.05
*P*-value	*t*0-*t*24-*t*48	>.05	>.05	

SOFA (points)	*t*0	13.0; 11.0–15.0	16.5; 15.0–18.0	>.05
	*t*24	13.0: 11.5–15.5	16.5; 14.3–18.0	>.05
	*t*48	14.0: 12.0–15.0	18.0; 9.0–18.0	>.05
*P*-value	*t*0-*t*24-*t*48	>.05	>.05	

SAPS II (points)	*t*0	60.5; 54.8–66.3	81.0; 72.3–89.3	>.05
	*t*24	73.0; 60.0–77.0	76.5; 66.0–84.5	>.05
	*t*48	63.0; 57.5–75.5	76.0; 71.0–90.0	>.05
*P*-value	*t*0-*t*24-*t*48	>.05	>.05	

Data are presented by median and interquartile range (Q1–Q3).

**Table 3:** Comparison of IL-6, TRX1, and MIF plasma levels in the volunteer, postoperative, and septic group at baseline.

	Healthy (*n* = 18)	Post-OP (*n* = 28)	Sepsis (*n* = 15)
IL-6 (pg/ml)	0.0; 0.0–0.8	216.7; 48.8–360.5	577.7; 521.9–583.2
*P*-value	Healthy versus Post-OP: *P* < .001***		
	Healthy versus Sepsis: *P* < .001***		
	Post-OP versus Sepsis: *P* <.001***		

TRX1 (ng/ml)	11.0; 10.0–12.1	11.3; 9.6–15.6	34.0; 19.7–57.7
*P*-value	Healthy versus Post-OP: *P* = .458		
	Healthy versus Sepsis: *P* < .001***		
	Post-OP versus Sepsis: *P* <.001***		

MIF (pg/ml)	161.6; 148.5–214.0	156.4; 115.1–398.7	1821.1; 412.2–3708
*P*-value	Healthy versus Post-OP: *P* = .954		
	Healthy versus Sepsis: *P* = .005**		
	Post-OP versus Sepsis: *P* < .001***		

Data are presented by median and interquartile range (Q1–Q3).
